# A time estimation task as a possible measure of emotions: difference depending on the nature of the stimulus used

**DOI:** 10.3389/fnbeh.2015.00143

**Published:** 2015-06-11

**Authors:** Auriane Gros, Maurice Giroud, Yannick Bejot, Olivier Rouaud, Sophie Guillemin, Corine Aboa Eboulé, Valeria Manera, Anaïs Daumas, Martine Lemesle Martin

**Affiliations:** ^1^Department of Neurology, University Hospital of Dijon and EA4184 of the University of BurgundyDijon, France; ^2^CoBTek (Cognition—Behaviour—Technologie), Nice Sofia-Antipolis University, Institut Claude PompidouNice, France; ^3^Resource and Research Memory Center, Hospital of DijonDijon, France; ^4^Dijon Stroke Registry, EA4184, University Hospital and Medical School of Dijon, University of BurgundyDijon, France; ^5^Department of Neurophysiology, University Hospital of DijonDijon, France

**Keywords:** emotional disorders, priming effect, skin conductance, time estimation, test

## Abstract

**Objective**: Time perception is fundamental for human experience. A topic which has attracted the attention of researchers for long time is how the stimulus sensory modality (e.g., images vs. sounds) affects time judgments. However, so far, no study has directly compared the effect of two sensory modalities using emotional stimuli on time judgments.

**Methods**: In the present two studies, healthy participants were asked to estimate the duration of a pure sound preceded by the presentation of odors vs. emotional videos as priming stimuli (implicit emotion-eliciting task). During the task, skin conductance (SC) was measured as an index of arousal.

**Results**: Olfactory stimuli resulted in an increase in SC and in a constant time overestimation. Video stimuli resulted in an increase in SC (emotional arousal), which decreased linearly overtime. Critically, video stimuli resulted in an initial time underestimation, which shifted progressively towards a time overestimation. These results suggest that video stimuli recruited both arousal-related and attention-related mechanisms, and that the role played by these mechanisms changed overtime.

**Conclusions**: These pilot studies highlight the importance of comparing the effect of different kinds on temporal estimation tasks, and suggests that odors are well suited to investigate arousal-related temporal distortions, while videos are ideal to investigate both arousal-related and attention-related mechanisms.

## Introduction

Time perception is fundamental for human experience; it is essential for everyday behavior and for understanding social interactions. Humans are able to estimate time with an amazing precision, an ability which has been hypothesized to rely on the existence of an internal clock (Gibbon, 1977). However, time judgments can become extremely inaccurate under the influence of emotions (Droit-Volet and Meck, 2007). Several studies have shown that when participants are emotionally aroused, they tend to overestimate time, that is to report that the duration of a stimulus is longer than what it actually is (see Droit-Volet and Gil, 2009, for a review). However, the valence of emotions (Leppänen et al., 2003) and the level of arousal (Gil and Droit-Volet, 2012) do play an important role leading either to a time overestimation or to a time underestimation depending on the emotional stimulus employed (Angrilli et al., 1997; see below).

A topic which has attracted the attention of researchers since long time is how stimuli presented in different sensory modalities can affect time estimations. For example, Penney and Tourret (2005) examined the differential effects of auditory and visual signals on clock speed and temporal memory, suggesting that the duration of visual stimuli is underestimated compared to that of auditory stimuli of equivalent duration (see also Zélanti and Droit-Volet, 2012). However, to our knowledge, no study has directly compared the effect of two sensory modalities using emotional stimuli on time judgments. Moreover, a number of previous studies employed auditory (sounds, music; Lambrechts et al., 2011 using IADS) or visual stimuli (images, videos; Noulhiane et al., 2007; Zélanti and Droit-Volet, 2012), but only one study has been conducted so far using olfactory stimuli, which are well known to activate the emotional system (Schreuder et al., 2014). One of the reasons of the limited use of olfactory stimuli is that in the classical paradigm (the temporal bisection task) participants are asked to estimate the duration of emotional stimuli. This paradigm is clearly not appropriate to investigate the effect of olfactory stimuli.

The aim of the present work was to compare the effect of emotionally arousing video stimuli and odors by using an implicit emotional priming paradigm that was developed in previous works (Gros et al., 2014). In the next sections we will first describe how time estimation works, by describing the most relevant models of the internal clock. Second, we will briefly describe how emotions affect time estimations. Third, we will describe the emotional priming paradigm and the Clock’n test, an emotional priming task based on olfactory stimuli that we recently developed.

### The Internal Clock Models

**Figure 1:**
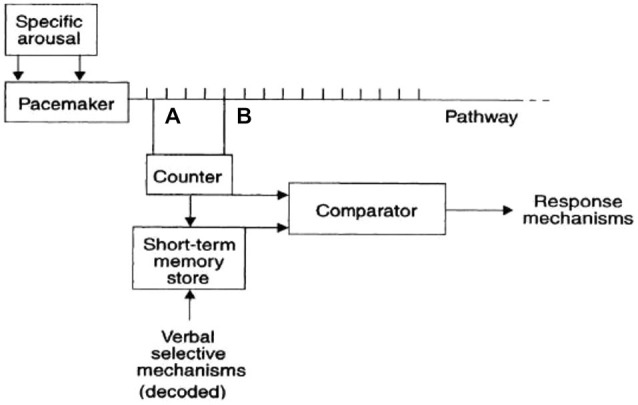
**The internal clock model proposed by Treisman and Temporal (1963)**.

Some researchers attempted to model our internal time-measuring mechanism as a sort of internal clock. In a seminal work, Treisman and Temporal (1963) proposed that the internal clock consists of an arousal-sensitive pacemaker, which sends continuously and constantly pulses to a counter. The model also involves a store of “reference” durations, and a comparator mechanism. Subjective time estimations derive from the comparison of values in the counter and in the store (see Figure 1). In this model, internal and external stimuli are supposed to modify the activity of the internal clock by modifying the activity of the peacemaker. For instance, arousing emotional stimuli are supposed to speed up the pacemaker, which sends more pulses to the counter, thus resulting in a subjective time overestimation.

**Figure 2:**
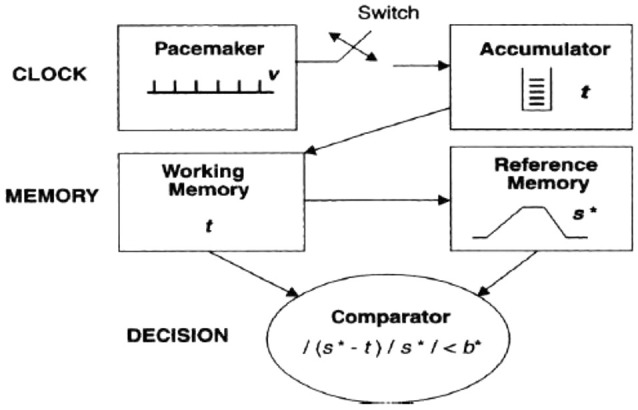
**The temporal information-processing model proposed by Gibbon et al. (1984)**.

Treisman’s internal clock model was remodeled by Gibbon’s scalar theory in 1977 and 1984. Gibbon’s model shares with Treisman’s model basic architecture (see Figure 2), but it introduced the idea of a switch, which can stop the pulse count and can be activated by attentional mechanisms. When the switch is activated, it prevents the pulses to enter into the accumulator, thus resulting in a time underestimation.

### Emotions and Time Estimation

Consistent with the internal clock models, studies conducted so far demonstrated that emotional stimuli can result either in a time overestimation, either in a time underestimation depending on the stimulus employed, and specifically depending on whether the emotional arousal component or the attentional component is prevailing (Lejeune, 1998; Elliott et al., 2000; Burle and Casini, 2001). The arousal-elicited time overestimation has been studied since the 1960s. Langer et al. (1961) were the first to demonstrate that danger stimuli, which are highly arousing, induced temporal overestimation. In 1984, Watts and Sharrock found converging results. Thayer and Schiff (1975) showed that the simple act of watching someone expressing an emotion caused an acceleration of the internal clock resulting in a time overestimation and, more recently, Noulhiane and colleagues (Noulhiane et al., 2007) showed a temporal overestimation during the presentation of emotional auditory stimuli compared to neutral stimuli. Similarly, Cocenas-Silva et al. (2011) showed that the duration of arousing music was overestimated. The same results were obtained employing visual stimuli, specifically pictures of emotional facial expressions (see Gil and Droit-Volet, 2012) and dynamic video clips of emotional expressions (Fayolle and Droit-Volet, 2014). Also, Lambrechts et al. (2011) showed that emotional content-bearing stimuli (IAPS pictures) were judged to last longer than content-deprived stimuli (gray squares). A recent study by Schreuder et al. (2014) employed olfactory stimuli associated with body postures to explore how arousal affected perceived duration in a time production task. They found that odors resulted in a time overestimation. However, skin conductance (SC) did not increase during the presentation of odors, which may suggest that the effect of olfactory stimuli on time estimation in their task was not mediated by arousal.

Recent studies have also confirmed the involvement of the attentional system in emotion-related time estimates. Angrilli et al. (1997), using emotional pictures, showed that the duration of highly arousing, unpleasant pictures (mutilated bodies) was overestimated, whereas the duration of highly arousing pleasant pictures (erotic scenes) was underestimated. Inversely, when employing low arousing pictures, the duration of unpleasant pictures was underestimated, while the duration of pleasant pictures was overestimated. The authors interpreted this opposite direction of the valence effect as a function of arousal as evidence that two different mechanisms are involved: an attention-driven mechanism for low arousal, and an emotion-driven mechanism for high arousal. Also, Droit-Volet et al. (2010) showed that the duration of musical excepts was underestimated compared to the duration of neutral sounds, possibly due to the fact that music captures the attentional system more consistently.

Zélanti and Droit-Volet (2012) have recently shown that children become more accurate in estimating the duration of sounds (a synthetic piano tone) earlier compared to what occurs for images (solid circle), a result which has been interpreted as evidence that visual stimuli engage the executive attentional system more consistently than auditory stimuli. However, no study, so far, has directly compared directly the effect of emotional stimuli presented in different sensory modalities on time estimations.

#### The Priming Paradigm and the Clock’n Test

The priming paradigm is ideal to explore emotional processing in an implicit way, and to minimize the role played by cognitive factors. The priming paradigm consists of presenting two stimuli sequentially, and observing the influence of the first (the prime) on the second (the target). Various studies have shown that the effect is generally obtained when less than 300 ms separate the appearance of the prime and the target (Hermans et al., 2001). Studies have shown that odors are particularly effective as priming stimuli, and that cognitive processes such as attention, reasoning and recall can be influenced by olfactory stimuli. Also, neuroimaging and EEG data (Kline et al., 2000) indicate that odors can affect the nervous system even without being consciously perceived.

Based on these observations, we recently developed the Clock’n test (Gros et al., 2014). In this test, participants have to estimate the duration of a neutral sound through a classical temporal bisection task. Critically, the presentation of the sound is preceded by the presentation of odors as priming stimuli, which generate emotional arousal, and thus a time overestimation.

#### The Present Work

The aim of the present work was to compare the results obtained by using different kinds of emotional priming stimuli, specifically odors and emotional video clips. In Study 1, participants were asked to perform a temporal bisection task estimating the duration of a target neutral sound. The target sound was preceded by the presentation of videos or odors, employed as priming stimuli. In order to verify that stimuli were effective in generating an emotional response, participant’s SC was measured before and during the task. Study 2 was designed to replicate the results of Study 1 using a different, randomized order of presentation of priming stimuli and target sounds.

## Study 1

### Material and Methods

#### Ethics Statement

Ethics approval was granted by the Ethics Committee Est I (France), and the study was labeled as non-interventional. A statement was made to the National Commission for Computing and Liberties by the Department of Clinical Research and Innovation by the Hospital of Dijon. Statement Number: 1758780v0, April 2014.

#### Participants

50 adults participated in this study (23 M, 27 F; age range: 55–95 years^1^; mean age: 75 years). They were all residents of the Burgundy region (France), right-handed, and their mother tongue was French. Their cognitive functions were assessed by means of the Folstein Mini Mental Examination (MMSE; Folstein et al., 1975), the Faux Pas test (Gregory et al., 2002) and the Hamilton Depression Rating Scale (Hamilton, 1980). Abnormal results to these tests represented non-inclusion criteria. Also, participants were not included if they had pathologies causing cognitive impairment (e.g., brain tumor, neurological disorder), olfactory impairments (anosmia, hyposmia, cacosmia) or auditory impairments (hypoaccousie, auditory hallucinations).

#### Tasks and Design

Participants were randomly assigned to one of two conditions (“olfactory stimuli” vs. “video stimuli”), making sure that the two groups were balanced for gender and age. In both groups, participants were initially administered with a time estimation task (phase 1). In the “olfactory stimuli” condition, participants performed a second time estimation task preceded by the presentation of olfactory stimuli as priming stimuli (phase 2). In the “video stimuli” condition, the second time estimation task was preceded by the presentation of video stimuli as priming stimuli (phase 2). Olfactory stimuli and video stimuli were counterbalanced for valence based on results of previous studies (see below). Specifically, for the odor condition, four stimuli had a positive valence, and three stimuli had a negative valence, while for the video condition three stimuli had a positive valence, and four stimuli had a negative valence. In both conditions during phase 2, participant’s SC was measured by means of an equipment of measuring electrodermal response to the Neurophysiology Department of Dijon.

#### Priming Stimuli and Target Sounds

##### Olfactory Priming Stimuli

The olfactory stimuli were part of the battery Biolfa (CCA Biodigital Amplifon, Paris, France). The battery comprises two sets of glass bottles containing 30 ml of odorant molecules dissolved in ethylene glycol dipropyl. The following seven odorants were selected: para cresyl (smell of horse manure), citronnella (lemongrass), cis-3 hexanol (clippings), l-carvone (mint), eugenol (nail clove), 1-octene-3ol (fungus), and vanillin (vanilla). The valence of each stimulus collected in a previous study (Gros et al., 2014) is reported in Table 1. For each valence (positive or negative), participants (75 subjects) used a 5-point scale (1 “very slightly or not at all”, 5 “extremely”) to rate the extent to which they felt a positive and a negative state as they were sniffing the odor.

**Table 1:** **Video and odor stimuli with level of positive valence and negative valence**.

Num stimulus	Film scene	Positive valence	Negative valence	Odor	Positive valence	Negative valence
1	The dinner game	3.89	1.11	Vanillin	3.56	0
2	Chucky ii	1.34	2.01	Eugenol	1.7	1.9
3	The lover	1.63	1.13	Citronnelle	2.76	2.23
4	Copycat	1.19	2.18	1Octene3-ol	2.82	3.22
5	Scream 2	1.74	1.95	Cis-3-hexanol	2.86	1.87
6	Misery	1.24	3.21	Para-cresyl	0.5	3.21
7	Ghost	3.88	1.16	Carvone	3.24	2.5

##### Video Priming Stimuli

The video stimuli were selected from the tool developed by Schaefer et al. (2010). We conducted pretests with more than 50 healthy participants, to select the films clips that were not traumatizing, but still able to generate a consistent electrodermal response. Seven films were selected: three films with positive valence and four with negative valence (see Table 1). In the original study, the valence (positive and negative) was assessed with a validated French translation (Gaudreau et al., 2006) of the Positive and Negative Affect Schedule (PANAS; Watson et al., 1988). For each of the 20 emotion-related words, participants used a 5-point scale (1: “very slightly or not at all”, 5: “extremely”) to rate the extent to which they felt each emotion as they were watching the film clip.

##### Target Sounds Employed for the Time Estimation Task

The auditory stimuli employed for the time estimation task were generated with the software PRATT (LF-ARX model). This is an algorithm for the sound analysis synthesis able to produce different voice components which has often been employed to explore the acoustic components associated to different emotions. Critically, the software allows generating pure sounds with the desired frequency and intensity. For the purposes of the present study, we selected a pure sound, which has the advantage of not being associated to any personal experience, as it does not exist in nature. The same sound was generated in seven different durations all shorter than 2 seconds (0.4, 0.6, 0.8, 1, 1.2, 1.4, and 1.6 s). Indeed, we know that for sounds longer than 2 s we can use other strategies, such as counting, to increase the time estimation accuracy (Burle and Casini, 2001).

#### Experimental Design and Procedure

The psychophysiological study took place in a temperature controlled and well ventilated room at the Exploration Lab Nervous System of Dijon (France). Participants were seated in a comfortable chair and their non-dominant hand was placed on a soft pillow. SC was measured in real-time via a MP100WSW hardware, and was recorded using a GSR100B amplifier and 6 mm inner diameter Ag/AgCl finger electrodes (TSD203) via the constant voltage (0.5V) technique. Electrodes were filled with conductive gel and placed on the second phalanx of the middle and the index finger of the non-dominant hand with non-caustic adhesive tape. Electrode positioning was in compliance with traditional recommendations (Fowles et al., 1981).

Skin conductance (SC) was measured before starting the task (in order to record a baseline, and to ensure that participants were not already emotionally aroused before starting the procedure) and during the task.

The experiment consisted of two phases occurring sequentially. In the first phase (phase 1) we determined a time judgment baseline for each participant, as duration judgments are known to vary consistently across individuals, and are affected by multiple factors (e.g., time of the day, age, sex, attention) (Lejeune, 1998; Bschor et al., 2004; Droit-Volet et al., 2007). In the second phase (phase 2) we explored whether the subjective time judgment varied with the presentation of olfactory or visual priming stimuli.

##### Phase 1: Subjective Time Estimation

As temporal distortions can be caused by several factors, including individual differences (Bschor et al., 2004; Droit-Volet et al., 2007), before starting the task we create a subjective time estimation baseline for each participant, by asking the participant to judge the duration of the same sounds presented later in the priming task.

Similarly we collected the basic electrodermal response of each participant because they can vary depending on many factors (such as age, sex, health status, etc.).

Participants were presented with a paper sheet with a 20 cm line with 3 anchor points indicating 0, 1, and 2 s. Before starting the time estimation task, participants were presented with two sounds of 1 s and 2 s, to give them standard reference points. Participants were then presented with seven sounds of 400, 600, 800, 1000, 1200, 1400, and 1600 ms in the following order: 1400, 600, 1200, 400, 800, 1000, and 1600 ms. The presentation order was the same for all participants. After each sound, participants were asked to bisect the line in front of them to indicate the estimated duration of the sound. These estimates were used as a baseline for the time estimation made in Phase 2.

##### Phase 2: Priming Task with Smell vs. Video Stimuli

Depending on the condition to which he/she was assigned (group 1 = odor Group 2 = video), participants were asked to either smell something or to watch a short video-clip. In the smell condition, participants were asked to smell an odor. After 300 ms from the odor presentation (Hermans et al., 2001), participants were presented with a target sound and asked to estimate its duration with the same line bisection procedure used in Phase 1. The time interval between two different trials was one and a half minutes and equivalent across conditions. The procedure was repeated 7 times, with 7 different odors and the 7 sounds employed in Phase 1 (presented in the same randomized order as in Phase 1). In the video condition, the procedure was exactly the same, but the smell stimuli were substituted by the video stimuli. Participants were not asked to provide ratings on the emotional priming stimuli because this would have focused their attention to the emotional aspects of the task.

### Data Analysis

In order to obtained an unbiased measure of time estimation, for each participant and for each stimulus we calculated the time warp as the difference between the time estimation provided in Phase 2 and the time estimation provided in Phase 1. Time warp was then submitted to a repeated-measure ANOVA with presentation order (from the 1st to the 7th presented stimulus) as a within-subject variable and condition (odor vs. video) as a between-subjects factor. The ANOVA linear contrast was also reported. SC was measured by means of the amplitude of the maximum peak shown in correspondence of the stimulus presentation. Also, SC was submitted to a repeated-measures ANOVA with presentation order (from the 1st to the 7th presented stimulus) as a within-subject variable and condition (odor vs. video) as between-subjects factor. The ANOVA linear contrast was also reported. Statistical analyzes were performed using SPSS 20.0 (IBM).

### Results

#### Time Estimation

The time warp for the different experimental conditions is reported in Table 2 (mean ± standard deviation).

**Table 2:** **Timewarp following an olfactory stimulus compared to the time warping after a video stimulus**.

Time warp after an olfactory stimulus (sec.)	Mean score ± SD	Time warp after a video stimulus (sec.)	Mean score ± SD
1. Odor 1	0.29±0.16	1. Film 1	−0.49±0.33
2. Odor 2	0.23±0.11	2. Film 2	−0.38 ±0.27
3. Odor 3	0.24±0.13	3. Film 3	−0.20±0.29
4. Odor 4	0.27±0.13	4. Film 4	−0.03±0.13
5. Odor 5	0.22±0.18	5. Film 5	−0.10±0.34
6. Odor 6	0.20±0.14	6. Film 6	−0.01±0.38
7. Odor 7	0.20±0.16	7. Film 7	0.20±0.37

**Figure 3:**
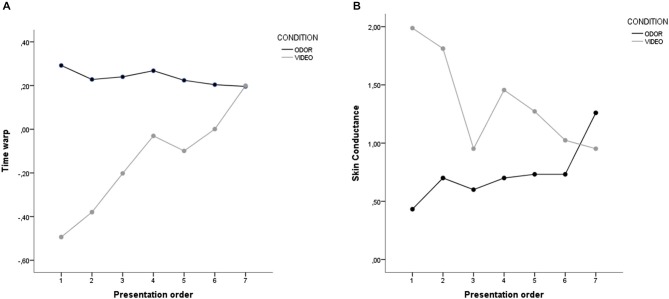
**Results of Study 1. (A)** Time warp and **(B)** skin conductance for the odor and the video condition. Stimuli on the abscissa are displayed by order of presentation.

ANOVA on time warp revealed a significant main effect of presentation order, with the last presented stimuli resulting in a higher time overestimation compared to the first presented stimuli (*F*_(6,288)_ = 9.58, *p* < 0.001). *Post hoc* comparisons (Bonferroni-corrected) revealed that the 1st presented stimulus resulted in a lower time overestimation compared to the 4th (*p* < 0.001), the 5th (*p* = 0.018), the 6th (*p* < 0.001), and the 7th (*p* < 0.001) presented stimuli. Also, a similar difference was found between the 2nd and the 4th (*p* < 0.001), 6th (*p* = 0.010), and 7th stimulus (*p* < 0.001). A main effect of condition was also found, with odor stimuli resulting in a higher mean time overestimation compared to video stimuli (*p* < 0.001). Critically, these main effects were moderated by a highly significant interaction between presentation order and condition (*p* < 0.001; see Figure 3A). To further explore this interaction, we performed two separates ANOVAs on time warp for the video and odor condition. Results revealed no significant effect of presentation order for the odor condition, thus suggesting that odor presentation resulted in a time overestimation independently of the order in which stimuli were presented (*F*_(6,144)_ = 1.49, *p* = 0.203). On the contrary, a highly significant effect of presentation order was found for the video condition (*F*_(6,144)_ = 18.16, *p* < 0.001; linear contrast: *F*_(1,24)_ = 72.77, *p* < 0.001), with time warp shifting progressively from a time underestimation to a time overestimation. In order to check whether the time warp change was driven by the stimulus presentation order and not by the video arousal, we recomputed the ANOVA ordering the stimuli based on the arousal. The linear contrast using arousal as a ordering variable was not statistically significant (*F*_(1,24)_ = 2.63, *p* = 0.118), thus suggesting than the presentation order was indeed the critical variable in generating the time warp shift. Furthermore, in order to check whether the time warp change was driven by the duration of the target sounds, we recomputed the ANOVA ordering the stimuli based on the target sound duration (from the shorter, 400 ms, to the longer, 1600 ms). The linear contrast using the duration of the target sounds as a ordering variable was not statistically significant (*F*_(1,24)_ = 1.25, *p* = 0.274), thus suggesting than the presentation order, and not the duration of the target sounds, was indeed the critical variable in generating the time warp shift.

Finally, in order to explore potential effects of the emotional valence of the priming stimuli (positive vs. negative), we compared the mean time warp observed in correspondence of positive and negative priming stimuli in both the video and the odor condition. ANOVA revealed no difference in time warp between positive and negative videos (*F*_(1,24)_ = 0.79, *p* = 0.384), nor between positive and negative odors (*F*_(1,24)_ = 0.04, *p* = 0.846).

#### Skin Conductance (SC)

ANOVA on SC revealed a significant main effect of presentation order (*F*_(6,288)_ = 9.58, *p* < 0.001), with the first presented stimuli associated to a higher SC compared to the last presented stimuli. *Post hoc* comparisons (Bonferroni-corrected) revealed that the 1st presented stimulus was associated to a higher SC compared to the 3rd (*p* < 0.001) and 6th (*p* = 0.003) presented stimuli. The 2nd presented stimulus showed a higher SC compared to the 3rd (*p* < 0.001), 5th (*p* = 0.025) and 6th (*p* < 0.001) stimuli. Finally, the 3rd stimulus had a higher SC compared to the 4th (*p* = 0.015) and the 7th stimuli (*p* = 0.001). A main effect of condition was also found, with odor stimuli resulting in a lower SC compared to video stimuli (*p* < 0.001). Critically, these main effects were moderated by a highly significant interaction between presentation order and condition (*p* < 0.001; see Figure 3B). To further explore this interaction, we performed two separate ANOVAs on time warp for the video and odor condition. Results revealed a significant effect of presentation order for the odor condition, with SC increasing linearly from the first to the last presented stimuli (*F*_(6,144)_ = 34.56, *p* < 0.001; linear contrast: *F*_(1,24)_ = 76.82, *p < 0.001)*. Interestingly, the opposite effect was found for video stimuli, with SC decreasing linearly from the first to the last presented stimuli, thus suggesting an emotion regulation effect deployed over time (*F*_(6,144)_ = 16.74, *p* < 0.001; linear contrast: *F*_(1,24)_ = 45.05, *p* < 0.001). In order to check whether the SC change was driven by the stimulus presentation order and not by the video arousal, we recomputed the ANOVA ordering the stimuli based on the arousal. The linear contrast using arousal as a ordering variable was not statistically significant (*F*_(1,24)_ = 0.02, *p* = 0.902), thus suggesting than the presentation order was indeed the critical variable in generating the SC reduction. Finally, in order to explore potential effects of the emotional valence of the priming stimuli (positive vs. negative), we compared the mean SC observed in correspondence of positive and negative priming stimuli in both the video and the odor condition. The ANOVA revealed no difference in SC between positive and negative videos (*F*_(1,24)_ = 1.05, *p* = 0.316), nor between positive and negative odors (*F*_(1,24)_ = 1.66, *p* = 0.210).

## Study 2

In Study 1, the order of presentation of the priming stimuli and the target sounds was the same for all the participants. For this reason, it is not possible to exclude that the effect we observed on time warp was due to the order of presentation of the priming stimuli (videos and odors), of the target sounds employed in the time estimation task, or of both the priming stimuli and the target sounds. Study 2 was designed to replicate the results of Study 1 employing a randomized order of presentation of the priming stimuli (random priming stimuli) and of the target sounds (random target sounds).

### Participants

41 adults participated in this study (20 M, 21 F; 55–95 years; mean age: 75 years). They were all residents of the Burgundy region (France), right-handed, and their mother tongue was French. Their cognitive functions were assessed by means of the Folstein Mini Mental Examination (MMSE; Folstein et al., 1975), the Faux Pas test (Gregory et al., 2002) and the Hamilton Depression Rating Scale (Hamilton, 1980). Abnormal results to these tests represented non-inclusion criteria. Also, participants were not included if they had pathologies causing cognitive impairment (e.g., brain tumor, neurological disorder), olfactory impairments (anosmia, hyposmia, cacosmia), or auditory impairments (hypoaccousie, auditory hallucinations).

### Methods and Procedures

Participants were randomly assigned to one of two groups, either the “random priming stimuli” group or the “random target sounds” group, making sure that participants in the two groups were balanced for gender and age. Similarly to Study 1, participants in both groups were then randomly assigned to either the “olfactory stimuli” or the “video stimuli” condition. Stimuli and procedure were identical to that of Study 1. The only difference compared to Study 1 was that the order of presentation of the priming stimuli (in the “random priming stimuli” condition) and the target stimuli (in the “random target sounds” condition) was fully randomized across participants. Specifically, participants in the “random priming stimuli” group were administered the target sounds for the time estimation task in the same order used for Study 1, but which stimulus represented the priming for each target was fully randomized across participants. In the “random target sounds” group, priming stimuli (videos and odors) were presented in the same order used in Study 1, but the order of presentation of the target sounds employed for the time estimation task was fully randomized across participants. SC was not measured.

### Data Analysis

In order to obtained an unbiased measure of time estimation, for each participant and for each stimulus we calculated the time warp as the difference between the time estimation provided in Phase 2 and the time estimation provided in Phase 1. Time warp in the two groups (“random priming stimuli” and “random target sounds”) was then submitted to repeated-measures ANOVAs with presentation order (from the 1st to the 7th presented stimulus) as a within-subject factor and condition (odor vs. video) as a between-subjects factor. The ANOVA linear contrast was also reported.

### Results

**Figure 4:**
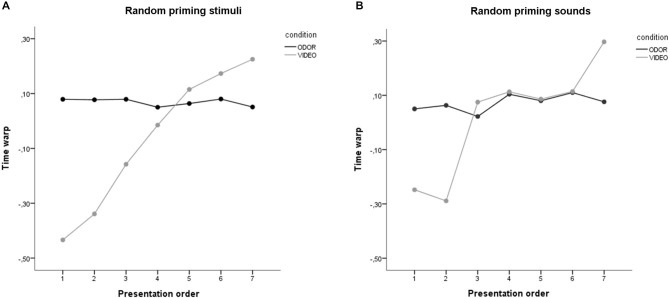
**Results of Study 2. (A)** Results of the random priming stimuli condition and **(B)** the random target sounds condition, for the odor and the video stimuli. Stimuli on the abscissa are displayed by order of presentation.

The time warp for the different groups and experimental conditions is reported in Figure 4. Specifically, panel *a* report the results for the “random priming stimuli” group, while panel *b* reports the results for the “random target sounds” group.

#### Random Priming Stimuli

Converging with the results of Study 1, ANOVA on time warp revealed a significant main effect of presentation order, with the last presented stimuli resulting in a higher time overestimation compared to the first presented stimuli (*F*_(6,114)_ = 12.61, *p* < 0.001). A main effect of condition was also found, with odor stimuli resulting in a higher mean time overestimation compared to video stimuli (*p* = 0.001). Critically, these main effects were moderated by a highly significant interaction between presentation order and condition (*p* < 0.001; see Figure 4A). To further explore this interaction, we performed two separates ANOVAs on time warp for the video and odor condition. Results revealed no significant effect of presentation order for the odor condition, thus suggesting that odor presentation resulted in a time overestimation independently of the number of stimuli presented (*F*_(6,60)_ = 0.63, *p* = 0.706). On the contrary, a highly significant effect of presentation order was found for the video condition (*F*_(6,54)_ = 12.92, *p* < 0.001; linear contrast: *F*_(1,9)_ = 28.48, *p* < 0.001), with time warp shifting progressively from a time underestimation to a time overestimation. This suggests that the order of presentation of the priming stimuli did not significantly affect the results of time warp found in Study 1. Furthermore, in order to check whether the time warp change was affected by the duration of the target sounds, we recomputed the ANOVA ordering the stimuli based on the target sound duration (from the shorter, 400 ms, to the longer, 1600 ms). The linear contrast using the duration of the target sounds as a ordering variable was not statistically significant (*F*_(1,24)_ = 1.29, *p* = 0.285), thus suggesting than the presentation order, and not the duration of the target sounds, was indeed the critical variable in generating the time warp shift.

#### Random Target Sounds

Converging with the results of Study 1 and of the random priming stimuli group, ANOVA on time warp revealed a significant main effect of presentation order, with the last presented stimuli resulting in a higher time overestimation compared to the first presented stimuli (*F*_(6,108)_ = 8.68, *p* < 0.001). The main effect of condition was not statistically significant (*p* = 0.113). Critically, the main effects were moderated by a highly significant interaction between presentation order and condition (*p* < 0.001; see Figure 4B). To further explore this interaction, we performed two separates ANOVAs on time warp for the video and odor condition. Results revealed no significant effect of presentation order for the odor condition, thus suggesting that odor presentation resulted in a time overestimation independently of the number of stimuli presented (*F*_(6,54)_ = 2.90, *p* = 0.123). On the contrary, a highly significant effect of presentation order was found for the video condition (*F*_(6,54)_ = 8.54, *p* < 0.001; linear contrast: *F*_(1,9)_ = 26.13, *p* = 0.001), with time warp shifting progressively from a time underestimation to a time overestimation. In order to check more directly whether the time warp in the video condition was affected by the duration of the target sounds, we recomputed the ANOVA using the target sounds duration as a repeated-measures factor. This showed no main effect of target sound duration on time warp (*F*_(6,54)_ = 1.56, *p* = 0.177). The linear contrast was also not statistically significant (*F*_(1,9)_ = 1.69, *p* = 0.226), thus confirming than the presentation order, and not the duration of the target sounds, was indeed the critical variable in generating the time warp shift observed in the video condition in both Study 1 and Study 2.

## Discussion

In the present two studies we compared the effect of emotional videos and arousing odors (used as priming stimuli) on a temporal bisection task. The results of Study 1 showed that the employed odors consistently activated the emotional/arousal system, as suggested by a consistent SC increase in correspondence of all the odor stimuli. No decrease of SC was observed overtime, thus suggesting that emotion regulation processes did not play a major role. Critically, the increase in arousal corresponded to a consistent time overestimation for all the seven odor stimuli. No effects of the priming stimulus valence were found nor for SC, nor for time warp. This arousal-dependent time overestimation is consistent with a number of previous studies. For instance, studies employing audio stimuli (Droit-Volet et al., 2010; Cocenas-Silva et al., 2011) showed that musical excerpts with high arousal produced a time overstimation, while the affective valence of the stimulus had little influence on time perception. Similar results have been found for stimuli presented in the visual modality (Nather et al., 2011; Gil and Droit-Volet, 2012; Fayolle and Droit-Volet, 2014), which systematically revealed that emotional pictures, which produced an increase in arousal, were systematically judged as longer compared to neutral pictures. A recent study by Schreuder et al. (2014) showed that olfactory stimuli caused a time overestimation (on stimuli of duration longer than one minute) even in absence of an increase in arousal, as measured by responses in SC and heart rate. This may suggest that the time overestimation effect that we found in correspondence of the olfactory stimuli may not entirely depend on the observed increase in arousal. However, it is also possible that results obtained using a time estimation based on long stimulus duration do not apply to short stimulus durations (less than two seconds), as different mechanisms may come into play (Droit-Volet and Meck, 2007; Gil and Droit-Volet, 2009). Future studies comparing stimuli of different durations may help to better clarify this issue.

The results of Study 1 showed that also the video stimuli generated a consistent emotional response, as confirmed by the SC measures, but this emotional reaction was progressively regulated over time, as shown by the drop in SC towards the end of the experiment. It should be noted that SC never reached the zero, suggesting that the last videos generated an emotional response, even if reduced compared to the first presented videos. Critically, these arousal changes had a strong correspondence with changes in time estimations. The first presented videos resulted in a time underestimation, which progressively shifted, towards the middle of the experiment, to a time overestimation. Study 2 replicated these results employing a randomized order of presentation of both priming stimuli (videos and odors) and target sounds employed in the time estimation task, thus suggesting that the shift from time underestimation to time overestimation was presentation-time dependent. No effects of the priming stimulus valence were found nor for SC, nor for time warp. Based on Gibbon’s clock scalar theory, we can hypothesize that at the beginning of the experiment attentional factors were prevailing: even if the pacemaker was accelerating and sending more pulses due to the emotional arousal, attentional mechanisms activated the internal switch, thus preventing the pulses to enter into the counter, and resulting in a time underestimation (Tse et al., 2004; Zakay, 2005; New and Scholl, 2009). This result is consistent with the studies showing that stimuli capturing attentional resources result in a time underestimation (e.g., Angrilli et al., 1997; Droit-Volet and Meck, 2007). Specifically, it has already been shown that complex visual stimuli consistently capture attentional resources (Droit-Volet et al., 2010). This supports the interpretation that the videos employed in the present study captured attentional resources, especially at the beginning of the task. After the presentation of some stimuli a habituation effect took place which, in our interpretation of the results, allowed to open the switch, and to shift progressively towards the classical emotion-elicited time overestimation. We believe that the shift occurred in the correspondence of the forth stimulus (and not the third video with the less level of SC, and, therefore, level of arousal) because attenuating the attentional factors takes some time. Thus, in summary, we believe that at the beginning of the experiment, attentional mechanisms activated the switch, which prevented the units of time to enter the counter. Emotion regulation meachisms reopened the switch, so the time units started to enter again into the counter, and after some time leaded to a time overstimation, due to emotional arousal generated by the stimuli. Further studies would be needed to corroborate this interpretation. Also, we did not find any effect of the duration of the target sounds (used in the time estimtion task), thus confirming that the results in the video condition are not explained by a clock speed type arousal effect. Rather, it is likely that attentional mechanisms played a major role (Angrilli et al., 1997).

The present results add to previous findings suggesting that when the arousal component is prevailing, participants tend to overestimate the duration of a stimulus, and that olfactory stimuli may be particularly suited to investigate the “pure” emotional response, with less involvement of attentional factors. The results also confirmed that visual stimuli recruit more consistently the attentional system. Critically, here we showed that attentional mechanisms are involved at the beginning of a task, but their importance in progressively reduced after repeated presentation of the emotional stimuli, thus suggesting that emotional video stimuli are useful to investigate both attentional mechanisms and emotional arousal.

It is important to note that, in our studies, we recruited only elderly participants. There is evidence that age plays a role on time estimates. For instance, using a time duration production task, Coelho et al. (2004) showed that time estimates were shorter for older than for younger participants, raising the possibility of a change in the rhythm of the internal clock with age. The tendency to underestimate time in older adults was also confirmed by Vanneste and Pouthas (1997) in duration estimation task, interpreted by the authors as possible evidence of a slowing down of the time processor during aging. Friedman and Janssen (2010) proposes several theories to explain this effect, including lower number of units entering the pacemaker, different response to emotional stimuli, and decrease in attentional resources. These age-related modifications in time estimates leave open the possibility that our results may not be exactly the same in a sample of younger adults. In future works, we aim at making a direct comparison with younger adults. If the age-related time underestimation is based on a change of the functioning of the internal clock, we expect that time underestimation will be less important in the younger adults compared to older adults. However, if the age-related underestimation is due to modifications in the attentional factors, we expect that, for the video condition, the time overestimation observed in the last video will be smaller.

### Limitations and Future Research Directions

It is widely recognized that emotions are complex sets of reactions consisting of three components: behavioral, cognitive-experiential-expressive, and physiological. The physiological component results in changes in the heart rate, electrodermal response (or skin conductance, SC), body temperature and respiratory rate. Most of the existing behavioral assessments for emotional experience are based on interviews and self-reports, or on the analysis of the social aspect of emotions (Bertoux et al., 2013; Millan and Bales, 2013; Parellada, 2013), which can capture the cognitive and the behavioral component of emotions, but they do not necessarily tap into the emotion physiological component. Furthermore, by assessing emotional experience explicitly, they are not easy to employ in patients with emotional awareness problems, such as alexitimia. We believe that the test described in the present study, focused on time estimations as an implicit measure of emotional arousal, may be successfully employed in several pathological populations to better understand the nature of their emotional deficits. In particular, the comparison of video and odor stimuli may give some indication of whether the deficit is more at the basic physiological level (no effect of the odor stimuli) or depends more on the attention/regulation component (no shift from time underestimation to time overestimation in the video condition).

Despite this, the study contributed to shed initial light on this complex topic, some limitations should be noted. First of all, we hypothesized that the time underestimation effect observed at the beginning of the video-task was due to the recruitment of attentional mechanism. However, some independent evidence is needed in order to corroborate this interpretation. Future studies directly manipulating attentional mechanisms—for instance, engaging participants in attention-demanding dual-tasks—could represent a good test. Second, the employed video-stimuli did not have the same duration, and it is possible that stimulus duration has an effect on time estimates, even if it is employed only as a prime stimulus. In order to control for this, we are running a normative study on 150 healthy participants, in which we are trying to replicate the present findings with video-stimuli of the same duration. Preliminary results suggest that stimulus duration is not a key variable in our time estimation task.

## Conflict of Interest Statement

The authors declare that the research was conducted in the absence of any commercial or financial relationships that could be construed as a potential conflict of interest.

## Footnotes

^1^In order to verify if the large age range affected our results, we run all the reported analyses by removing the seven participants older than 75 years. As all the reported results and significance were equivalent, we decided to keep all the participants in the sample.
